# A Home-Based Type 2 Diabetes Self-Management Intervention in Rural Guatemala

**DOI:** 10.5888/pcd14.170052

**Published:** 2017-08-10

**Authors:** David Flood, Jessica Hawkins, Peter Rohloff

**Affiliations:** 1 Wuqu’ Kawoq, Santiago Sacatepéquez, Sacatepéquez, Guatemala; 2 Medicine Pediatric Residency Program, University of Minnesota, Minneapolis, Minnesota; 3 Division of Global Health Equity, Brigham and Women’s Hospital, Boston, Massachusetts

## Abstract

**Introduction:**

Diabetes self-management education (DSME) is a fundamental element of type 2 diabetes care. Although 75% of adults with diabetes worldwide live in low-income and middle-income countries (LMICs), limited DSME research has been conducted in LMICs. The objective of this study was to evaluate a home-based DSME intervention in rural Guatemala.

**Methods:**

We conducted a prospective study of a DSME intervention using a quasi-experimental, single-group pretest–posttest design. We enrolled 90 participants in the intervention, which consisted of 6 home visits (May 2014–July 2016) conducted by a diabetes educator using a curriculum culturally and linguistically tailored to rural Mayan populations. Primary outcomes were changes in mean hemoglobin A1c (HbA1c) and mean systolic and diastolic blood pressure at baseline and at 12 months. Secondary outcomes were diabetes knowledge and self-care activities at baseline and intervention completion.

**Results:**

HbA1c decreased significantly from baseline to 12 months (absolute mean change, −1.5%; 95% confidence interval [CI], −1.9% to −1.0%; *P* < .001). Systolic blood pressure also improved significantly at 12 months (−6.2 mm Hg; 95% CI, −10.1 to −2.2 mm Hg; *P* = .002); changes in diastolic blood pressure were not significant (−1.6 mm Hg; 95% CI, −3.9 to −0.7 mm Hg; *P* = .17). We also found significant improvements in diabetes knowledge and self-care activities from baseline to intervention completion.

**Conclusion:**

DSME interventions can be successfully delivered in a setting with an underresourced health system, high poverty rate, and unique cultural characteristics like Mayan Guatemala. Our findings point to the need for more DSME research in resource-limited settings globally.

**MEDSCAPE CME**

Medscape, LLC, is pleased to provide online continuing medical education (CME) for this journal article, allowing clinicians the opportunity to earn CME credit.

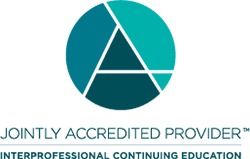
In support of improving patient care, this activity has been planned and implemented by Medscape, LLC, and *Preventing Chronic Disease*. Medscape, LLC, is jointly accredited by the Accreditation Council for Continuing Medical Education (ACCME), the Accreditation Council for Pharmacy Education (ACPE), and the American Nurses Credentialing Center (ANCC), to provide continuing education for the healthcare team.

Medscape, LLC, designates this Journal-based CME activity for a maximum of 1.00 *AMA PRA Category 1 Credit(s)™*. Physicians should claim only the credit commensurate with the extent of their participation in the activity.

All other clinicians completing this activity will be issued a certificate of participation. To participate in this journal CME activity: (1) review the learning objectives and author disclosures; (2) study the education content; (3) take the post-test with a 75% minimum passing score and complete the evaluation at http://www.medscape.org/journal/pcd; (4) view/print certificate.

**Release date:** August 10, 2017; **Expiration date:** August 10, 2018

**Learning Objectives**

Upon completion of this activity, participants will be able to:

1. Evaluate changes in hemoglobin A1c level after a diabetes self-management education (DSME) intervention among persons with type 2 diabetes in a low-resource global setting, based on a prospective, quasi-experimental, single-group study in Mayan Guatemala
2. Determine changes in blood pressure after a DSME intervention among persons with type 2 diabetes in a low-resource global setting, based on a prospective, quasi-experimental, single-group study in Mayan Guatemala
3. Compare changes in diabetes knowledge and self-care activities after a DSME intervention among persons with type 2 diabetes in a low-resource global setting, based on a prospective, quasi-experimental, single-group study in Mayan Guatemala

**EDITOR**

Ellen Taratus, MS

Editor, *Preventing Chronic Disease*

Disclosure: Ellen Taratus, MS, has disclosed no relevant financial relationships.

**CME AUTHOR**

Laurie Barclay, MD

Freelance writer and reviewer, Medscape, LLC

Disclosure: Laurie Barclay, MD, has disclosed the following relevant financial relationships:

Owns stock, stock options, or bonds from: Alnylam; Biogen; Pfizer Inc.

**AUTHORS**

David Flood, MD, MSc

Wuqu' Kawoq, Santiago Sacatepéquez, Sacatepéquez, Guatemala; Medicine Pediatric Residency Program, University of Minnesota, Minneapolis, Minnesota

Disclosure: David Flood MD, MSc, has disclosed no relevant financial relationships.

Jessica Hawkins, BA

Wuqu' Kawoq, Santiago Sacatepéquez, Sacatepéquez, Guatemala

Disclosure: Jessica Hawkins, BA, has disclosed no relevant financial relationships.

Peter Rohloff, MD, PhD

Wuqu' Kawoq, Santiago Sacatepéquez, Sacatepéquez, Guatemala; Division of Global Health Equity, Brigham and Women's Hospital, Boston, Massachusetts

Disclosure: Peter Rohloff, MD, PhD, has disclosed no relevant financial relationships.

## Introduction

Chronic, noncommunicable diseases such as diabetes are the leading cause of death globally (1). Of the 415 million adults with diabetes worldwide, 75% live in low-income and middle-income countries (LMICs) (2). However, the health systems of many LMICs are ill-equipped to deliver high-quality diabetes services (3).

Guatemala is a lower–middle-income Central American nation with a large rural and Maya indigenous population. Of a total population of 15 million, approximately 760,000 adults in Guatemala have diabetes. Despite limited epidemiologic data on diabetes in Guatemala (4,5), modeling studies suggest an age-adjusted national prevalence of 11.5% among men and 14.0% among women (6).

Lifestyle education and support is a fundamental element of diabetes care. Yet a striking contrast exists between the large burden of diabetes in LMICs and the small fraction of diabetes behavioral research conducted in these settings. In high-income countries, a robust literature supports the effectiveness of diabetes self-management education (DSME) interventions deployed through various delivery methods, training levels of personnel, and cultural specificity (7–9). In LMICs, however, only a few studies have investigated this topic (10–12), and the role of DSME interventions is unclear in settings with underresourced health systems, high poverty rates, and unique cultural characteristics, such as Maya Guatemala.

The objective of this study was to evaluate glycemic control and blood pressure outcomes of a small, home-based DSME intervention tailored to indigenous Maya adults with type 2 diabetes in rural Guatemala and implemented by a nongovernmental organization.

## Methods

This research was conducted by Wuqu’ Kawoq (www.wuqukawoq.org), a Guatemalan medical nongovernmental organization, in 4 municipalities in central Guatemala. The population in these municipalities is predominantly indigenous Maya in terms of language, culture, and dietary and work patterns. High-quality clinical diabetes care or diabetes education is largely unavailable in these areas (13). The first educational visit (and enrollment) occurred on May 3, 2014, and the last educational visit on July 16, 2016. 

### Study design and sample

This was a prospective study of a home-based DSME intervention using a quasi-experimental, single-group pretest–posttest design. It was approved by the institutional review boards of Wuqu’ Kawoq and Partners Healthcare, Boston. Participants were recruited from Wuqu’ Kawoq’s diabetes clinics. To be included in the study, participants were required to be 1) existing patients who had an HbA1c greater than 8.0% or had complications of diabetes or 2) patients newly presenting for care. The study had no exclusion criteria. No person meeting inclusion criteria was not enrolled. The intervention was incorporated into the ongoing standard of care at Wuqu’ Kawoq in April 2014, and participants were enrolled on a rolling basis. The sample in this study consisted of all participants who had been enrolled for at least 1 year when analysis began in September 2016. In addition to the home visits, all participants received free clinical care as detailed elsewhere (14), consisting of regular provider visits in the clinic, laboratory testing, treatment of hyperglycemia (including insulin), blood pressure management, and primary care coordination for specialty referrals.

### Formative research and intervention

In 2010, our group conducted a needs assessment on adult type 2 diabetes in rural Guatemala (15). We found that indigenous people with diabetes in this setting had limited understanding of the causes, chronicity, and complications of the disease. Education levels were low, and lack of social and family support were key barriers to making dietary and lifestyle changes. Many people with diabetes also described the financial challenges in carrying out dietary recommendations, such as increasing consumption of fruits and vegetables. In 2012, based on formative work and focus groups, we adapted a cardiovascular disease curriculum for use in low-literacy, Mayan-speaking populations based on a Guatemalan version of the US National Heart, Lung, and Blood Institute’s *Salud Para Su Corazón* (Health for Your Heart) community health worker model for Latinos (16,17). In late 2013, we tested the curriculum’s cultural acceptability and iterated the intervention based on a pilot with 10 people with diabetes.

This curriculum formed the basis of the education intervention in this study. In addition to general topics on cardiovascular disease, we emphasized the curriculum’s diabetes-related themes and offered participants practical strategies for implementing exercise routines and adhering to a diabetes diet in the rural Guatemalan setting where carbohydrate-rich corn tortillas are a dietary staple and an economical source of calories. Other adaptations included requesting family participation in education sessions, conducting the intervention in an individual home-based format rather than in a group format, minimizing use of written text, and integrating locally relevant drawings and props.

This intervention was delivered by a diabetes educator as a series of 6 home visits conducted Monday through Saturday during the day. Visits were planned as a weekly visit for the first month, a fifth visit at 3 months, and a sixth and final visit at 6 months. Visits could be rescheduled to account for a participant’s cancellation or unavailability. Sessions 1 through 3 focused on a theme (diet, exercise, or medical management), and in sessions 4 through 6, the diabetes educator reviewed progress and coached the patient through barriers relating to each theme. A full-time, bilingual (Spanish-Mayan) diabetes educator, who had a one-year postsecondary auxiliary nursing degree, delivered the sessions. The diabetes educator received more than 100 hours of training in the form of formal didactic sessions and clinic shadowing and jointly conducted home visits with clinical providers during the training period. The diabetes educator placed a brief note into the electronic health record (EHR) after each encounter; the note included information on themes discussed during the visit, visit duration, and the presence of family members. Clinical providers reviewed these notes with participants during monthly clinic visits to ensure intervention fidelity and reinforce educational themes.

### Measures

The primary clinical outcomes were glycemic control as assessed by mean hemoglobin A1c (HbA1c) and blood pressure as assessed by mean systolic and diastolic blood pressure. These clinical data were extracted from EHRs at 3 points: baseline, 6 months after enrollment, and 12 months after enrollment. HbA1c testing was conducted by using point-of-care devices (Quo-Lab, EKF Diagnostics; A1CNow+, Bayer, and PTS Diagnostics). Blood pressure was measured with standard manual blood pressure cuffs purchased from our local medical supply company (www.casamedica.com.gt). Given the study’s pragmatic nature and the variability in patients’ rescheduling, we defined “baseline” as 5 months before enrollment until 1 month after enrollment; at 6 months and 12 months, we allowed the nearest available data point within a 3-month window. At baseline only, we also extracted the following information for each participant from our EHRs: age, sex, time since diabetes diagnosis, selected prescriptions being taken (the following 4: metformin, sulfonylurea, insulin, and angiotensin-converting enzyme [ACE] inhibitors), and height and weight. We calculated body mass index (BMI) as weight in kilograms divided by height in meters squared and categorized overweight as a BMI of 25.0 or more (18). We did not assess changes in weight because of insufficient data.

Secondary outcomes were diabetes knowledge as measured by the 24-item Diabetes Knowledge Questionnaire (19) and diabetes self-management as assessed by selected culturally relevant questions from the 12-item Summary of Diabetes Self-Care Activities measured at baseline and at completion of the final home visit (20). We professionally translated each instrument from the previously validated Spanish versions to Kaqchikel, the local Mayan language, and then refined the translations during a 3-month pilot stage. At enrollment, participants also completed a questionnaire documenting maternal language, education, marital status, history of diabetes education, and household financial difficulties. The 41-item baseline questionnaire, including items from the 2 surveys, was administered orally because we anticipated high rates of illiteracy and because Mayan Kaqchikel and K’iche’ are primarily spoken (rather than written) languages in the participating communities.

### Data analysis

We used Stata version 13.0 (StataCorp LLC) for all analyses. We used descriptive statistics to summarize data on participants’ visits and demographic characteristics. To assess primary clinical outcomes, we constructed separate linear mixed models using Stata’s *mixed* function with robust standard errors for HbA1c, systolic blood pressure, and diastolic blood pressure. Fixed effects in all 3 models included age, time, and an interaction term between time and an indicator variable for whether the participant had received clinical care for less than 3 months before enrollment in the intervention. This interaction term was used to control for the effects of pharmacotherapy on the primary outcomes, as high-quality clinical diabetes care was not widely available in our setting. We modeled time as a categorical variable (0 months, 6 months, 12 months), which required no underlying assumptions about the function of the outcome variable over time. Random effects were used to account for within-subject correlation. We calculated outcomes as changes from baseline using the *margins* function.

We used the Wilcoxon signed rank test to compare outcomes for diabetes knowledge and diabetes self-management at the first home visit and last home visit. We compared participants with missing HbA1c measurements or survey data using 2-tailed Student *t* tests and the Fisher exact test. 

## Results

Ninety participants were enrolled in the study (Table 1). The sample consisted primarily of women (82%). The maternal language of most (75%) participants was Mayan, and overall educational attainment was low (median of 2 years of schooling completed). Few individuals had previously received formal diabetes education (9%), and approximately two-thirds had some difficulty meeting household financial needs. At enrollment, most (90%) participants were prescribed metformin, and 32% were prescribed insulin. Glycemic control was poor; mean HbA1c was 9.9% (standard deviation [SD]), 1.8%), and only 11% of participants had an HbA1c less than 8.0%. More than 70% of participants were overweight.

**Table 1:** Baseline Characteristics of Participants (N = 90) in a Home-Based Diabetes Self-Management Education Intervention in Rural Guatemala, 2014–2016^a^

Characteristic	No. of Participants With Data	Value^b^
**Age, mean (SD), y**	90	53.8 (12.3)
**Female, %**	90	82
**Time since diabetes diagnosis, median (IQR), y**	88	8 (4–14)
**Maternal language, %**		
Kaqchikel Mayan	90	63
K’iche’ Mayan		12
Spanish		24
**Education completed, median (IQR), y**	88	2 (0–5)
**Marital status, %**		
Married or partnered	89	80
Single, divorced, or widowed		20
**Previously received diabetes education, %**	90	9
**Difficulty paying for household expenses, %**		
At times	90	33
Often		30
**Prescriptions being taken, %**		
Metformin	90	90
Sulfonylurea		54
Insulin		32
ACE inhibitor		32
**HbA1c**		
Mean (SD)	89	9.9 (1.8)
Measure <8.0, %		11
**Blood pressure **		
Systolic, mean (SD), mm Hg	90	126 (21)
Diastolic, mean (SD), mm Hg		75 (10)
Has <140 mm Hg systolic and <90 mm Hg diastolic, %		72
**Body mass index,^c^ kg/m^2^**		
Median (IQR)	85	26.8 (24.7–29.3)
≥25.0, %		71
≥30.0, %		22

Abbreviations: ACE, angiotensin-converting enzyme; HbA1c, hemoglobin A1c; IQR, interquartile range; SD, standard deviation.

a Data on maternal language, education, marital status, history of diabetes education, and household financial difficulties were self-reported by participants through a brief orally administered questionnaire. All other data were extracted from electronic health records. Not all participants answered all questions, and not all data were available in the electronic health record. Percentages may not add to 100 because of rounding.

b Continuous variables that had normal distributions described as mean (SD); variables that had nonnormal distributions as median (IQR).

c National Heart, Lung, and Blood Institute (18).

Of the 90 participants, 71 (79%) completed all 6 visits in a median of 9.2 (interquartile range [IQR], 8.0–12.1) months. Seventy-nine (88%) participants completed at least 4 visits. Diabetes educators spent an average of 115 (SD, 42) minutes with participants per visit, or 10.0 (SD, 4.0) hours of mean contact time per participant during the study period. At least 1 family member participated in 39% (185 of 471) of home visits.

HbA1c data were available for 89 participants at baseline, 83 participants at 6 months, and 77 participants at 12 months. Mean (95% confidence interval [CI]) HbA1c was 9.9% (9.5%–10.3%) at baseline, 8.2% (7.8%–8.6%) at 6 months, and 8.4% (8.0%–8.8%) at 12 months. Mean HbA1c decreased significantly from baseline to 6 months (estimated absolute mean change, −1.7%; 95% CI, −2.2% to −1.2%; *P* < .001) and from baseline to 12 months (estimated absolute mean change, −1.5%; 95% CI, −1.9% to −1.0%; *P* < .001). Systolic blood pressure also declined significantly from baseline to 6 months (estimated mean change, −6.3 mm Hg; 95% CI, −10.2 to −2.4 mm Hg; *P* < .001) and from baseline to 12 months (estimated mean change, −6.2 mm Hg; 95% CI, −10.1 to −2.2 mm Hg; *P* = .002). However, changes in diastolic blood pressure were not significant from baseline to 6 months (estimated mean change, −1.3 mm Hg; 95% CI, −3.5 to 1.1 mm Hg; *P* = .27) or at 12 months (estimated mean change, −1.6 mm Hg; 95% CI, −3.9 to −0.7 mm Hg; *P* = .17).

When we compared the baseline HbA1c measurements of participants with missing data at 6 months or 12 months with the baseline HbA1c measurements of those who were not missing data at those times, we found no significant differences. The median time between baseline and midpoint HbA1c measurements was 214 days (IQR, 181–273 days) and between 6 months and completion was 195 days (IQR, 153–245 days).

Among the 71 participants who completed 6 visits and for whom we had complete survey data on the secondary outcomes of diabetes knowledge and diabetes self-management, we found significant improvements in diabetes knowledge and various self-care measures (Table 2). We found no differences in age, sex, baseline HbA1c, or baseline blood pressure between the 71 participants with complete survey data and the 19 participants with missing data.

**Table 2:** Secondary Survey Outcomes of Diabetes Knowledge and Self-Care Among 71 Participants Who Completed All 6 Visits in a Home-Based Diabetes Self-Management Education Intervention in Rural Guatemala, 2014–2016^a^

Metric	Baseline Median (IQR)	Visit 6 Median (IQR)	*P* Value^b^
**Diabetes Knowledge Questionnaire, no. of questions answered correctly^c^**	13 (9–15)	21 (18–23)	<.001
**Diabetes self-care measures,^d^ no. of days performed in most recent week**			
Followed healthful eating plan	0 (0–4)	5 (4–7)	<.001
Participated in at least 30 minutes of physical activity	0 (0–3)	4.5 (2–7)	<.001
Checked feet	2.5 (0–7)	6 (3–7)	.002
Took medications as recommended	5 (0–7)	7 (6–7)	<.001

Abbreviation: IQR, interquartile range.

a Ninety participants were enrolled in the intervention.

b Determined by Wilcoxon signed rank test.

c Of 24 questions in the Diabetes Knowledge Questionnaire (19).

d Assessed by selected culturally relevant questions from the Summary of Diabetes Self-Care Activities (20), measured at baseline and at completion of the sixth and final home visit. Two questions, about smoking and whether a participant knew what a carbohydrate was, were excluded from analysis because no participants smoked at baseline and because “carbohydrate” may not have been meaningfully translated into Mayan.

## Discussion

This was a quasi-experimental, single-group pretest–posttest study of a DSME intervention in rural Guatemala that found significant improvement in participants’ glycemic control and systolic (but not diastolic) blood pressure at 12 months. Secondary outcomes of diabetes knowledge and selected self-care activities also improved significantly during the intervention.

The decrease of −1.5% (95% CI, −1.9% to −1.0%) in HbA1c at 12 months found in our study compares favorably with related DSME interventions in high-income countries. A Cochrane meta-analysis of culturally appropriate education for people with type 2 diabetes calculated improvements in HbA1c of −0.2% (95% CI, −0.3% to −0.04%) at 12 months (7). As in other reviews of type 2 diabetes behavioral interventions (8,9), the Cochrane meta-analysis did not include studies conducted outside of high-income countries. There is a dearth of diabetes lifestyle research conducted in LMICs (10–12). Our study adds to the small volume of DSME research, nearly all quasi-experimental, that has been conducted in LMICs with high mortality rates (10). To our knowledge, our study is only the second DSME study to originate from Guatemala (21). Additionally, the absence of DSME studies in LMICs complicates the comparison between our intervention and those in the Cochrane review because studies conducted in high-income countries may examine populations that have lower baseline HbA1c values and thus may have smaller modest effect sizes.

Our study has implications for diabetes lifestyle interventions in low-resource global settings. For example, a meta-analysis of DSME studies conducted in high-income countries concluded that education intensity was a powerful predictor of effect, and interventions offering 10 or fewer hours of contact had minimal benefit (9). However, it is unclear if this finding holds outside of high-income settings. The interaction between intervention intensity and benefit is particularly important in settings such as rural Guatemala where resources must be judiciously allocated. Although we did not consider costs in our study, we are interested in examining intensity and cost-effectiveness in future studies.

Two further considerations arising from this study concern the type of delivery personnel and the role of familial support. The diabetes educator in our study was a health professional with a 1-year nursing degree rather than a community health worker. Although many high-quality trials from the United States have supported their role in diabetes education interventions (8), community health workers were not used in our study because of the wide availability of indigenous auxiliary nurses on the labor market and our institutional experience, shared by others elsewhere (22), that has shown that Maya people with diabetes prefer professional health workers. However, such contextual factors may not apply to other settings.

Despite our intervention’s emphasis on involving family members to generate social support for participants, family attendance during home visits was modest, with only 39% of visits registering family participation. Anecdotally, family members frequently commented on the time burden of participating in home visits. Despite the salience of family support that has emerged from qualitative investigations of type 2 diabetes (14,23), our results suggest that alternative forms of family engagement, including incentives or visits outside of working hours, should be explored.

The primary strength of this study concerns the practical challenges that were overcome to successfully deploy a DSME intervention in rural Guatemala. For example, HbA1c testing was not available in local laboratories, so we used point-of-care devices for HbA1c monitoring (14). We also used an EHR system to collect data and monitor intervention fidelity even though home visits were carried out in remote villages. Other strengths of this study include a follow-up period of 12 months, which is a reasonably long duration relative to other DSME studies performed in LMICs (10,11), a low dropout rate (88% of participants completed at least 4 of the 6 scheduled home visits), and the degree to which our intervention was adapted to Maya populations.

Our study is subject to several limitations and weaknesses. First, because we did not use an experimental design with a control group, our results are subject to residual confounding. One potential confounder is the degree to which the reconstitution of diabetes medical care in resource-limited settings can lead to dramatic glycemic improvements, limiting interpretation of the role of education or social interventions (24). Although our model included an indicator variable representing a clinical lead-in period of 3 months, we may not have adequately controlled for the effects of care reconstitution. Viewed alongside the paucity of diabetes education research performed in LMICs, this limitation suggests the need for randomized controlled trials of DSME in these countries. At the same time, randomized controlled trials of DSME are unlikely to be conducted in every population group around the world, so it is imperative that implementers also share their qualitative and quantitative experiences with diabetes education in LMICs.

Second, we did not formally validate the psychometric properties of the survey instruments in Kaqchikel, the local language. We addressed this issue by selecting instruments previously validated in Spanish, professionally translating them into Mayan, and then iterating them during the pilot period. Third, as in our underlying clinical diabetes program (14) and other community-based chronic disease interventions (23,25,26), we enrolled a disproportional number of women in our intervention. Such female predominance reflects a failure of chronic disease programs globally, including our own, to identify and overcome the barriers men confront in receiving care (27). This topic has emerged as a research and programmatic priority for our institution. Fourth, high rates of elevated BMI (especially in women) have been reported in rural Guatemala (4,28,29). In our sample, a substantial proportion of participants (70%) also were overweight, yet we did not measure or target weight loss. We plan to prioritize weight in future iterations of the intervention.

A final limitation concerns the external validity of our findings. Our sample size was small, and all participants received care in the same clinical environment of a private, nonprofit free clinic. The feasibility and effectiveness of diabetes education in other environments (eg, a public health center or fee-for-service private clinic) is not known. Additionally, given chronic underfunding of the Guatemalan public health system, it seems that DSME interventions are most likely to be scaled and sustained by the private sector, although it is unclear if patients are willing to pay for this service. Our small sample size should be viewed as a limitation in the context that few published examples exist of comprehensive diabetes programs serving comparably poor, rural, and marginalized populations with a clinical volume similar to or greater than ours (30,31). Lastly, this intervention was tailored to Maya people with type 2 diabetes in rural Guatemala and should be generalized cautiously. Aspects of our intervention that may be useful in other resource-limited settings are prioritization of indigenous languages, home-based education delivery, and incorporation of family members.

This study’s findings support the role of DSME in low-resource settings globally and show the need for more and higher-quality diabetes behavioral research in LMICs.

## Post-Test Information

To obtain credit, you should first read the journal article. After reading the article, you should be able to answer the following, related, multiple-choice questions. To complete the questions (with a minimum 75% passing score) and earn continuing medical education (CME) credit, please go to http://www.medscape.org/journal/pcd. Credit cannot be obtained for tests completed on paper, although you may use the worksheet below to keep a record of your answers.

You must be a registered user on http://www.medscape.org. If you are not registered on http://www.medscape.org, please click on the “Register” link on the right hand side of the website.

Only one answer is correct for each question. Once you successfully answer all post-test questions, you will be able to view and/or print your certificate. For questions regarding this activity, contact the accredited provider, CME@medscape.net. For technical assistance, contact CME@medscape.net. American Medical Association’s Physician’s Recognition Award (AMA PRA) credits are accepted in the US as evidence of participation in CME activities. For further information on this award, please go to https://www.ama-assn.org. The AMA has determined that physicians not licensed in the US who participate in this CME activity are eligible for *AMA PRA Category 1 Credits™*. Through agreements that the AMA has made with agencies in some countries, AMA PRA credit may be acceptable as evidence of participation in CME activities. If you are not licensed in the US, please complete the questions online, print the AMA PRA CME credit certificate, and present it to your national medical association for review.

## Post-Test Questions

### Study Title: A Home-Based Type 2 Diabetes Self-Management Intervention in Rural Guatemala

#### CME Questions

1. You are advising a clinic in a low-resource area regarding the feasibility and likely outcomes of diabetes self-management education (DSME). According to the prospective, quasi-experimental, single-group study in Mayan Guatemala by Flood and colleagues, which one of the following statements about changes in hemoglobin A1c (HbA1c) level after a DSME intervention among persons with type 2 diabetes in a low-resource global setting is correct?
  - HbA1c decreased significantly by 1.5 from baseline to 12 months (95% confidence interval [CI], −1.9 to −1.0; *P *<.001)
  - Decrease in HbA1c was considerably less than that seen with similar DSME interventions in high-income countries (HICs)
  - Findings for HbA1c after a DSME intervention confirmed those of numerous previous studies in Guatemala and other low- and middle-income countries
  - In this study, HbA1c testing was performed in local laboratories
2. On the basis of the prospective, quasi-experimental, single-group study in Mayan Guatemala by Flood and colleagues, which one of the following statements about changes in blood pressure after a DSME intervention among persons with type 2 diabetes in a low-resource global setting is correct?
  - Systolic blood pressure did not change significantly from baseline to 6 months after the DSME intervention
  - Systolic blood pressure decreased significantly between baseline and 12 months (−6.2 mm Hg; 95% CI, −10.1 to −2.2 mm Hg; *P *=.002)
  - Diastolic blood pressure decreased significantly from baseline to 6 months and from baseline to 12 months
  - Decreases in blood pressure correlated significantly with weight loss
3. According to the prospective, quasi-experimental, single-group study in Mayan Guatemala by Flood and colleagues, which one of the following statements about changes in diabetes knowledge and self-care activities after a DSME intervention among persons with type 2 diabetes in a low-resource global setting would be correct?
  - Diabetes Knowledge Questionnaire score improved from 15 of 24 questions answered correctly before to 18 of 24 questions answered correctly after the intervention, which was not significant
  - Following a healthful eating plan improved from a median of 2 days per week before to 3 days per week after the intervention
  - Participation in physical activity did not improve significantly after the intervention
  - Taking medications as recommended improved from a median of 5 days per week before the intervention to 7 days per week after the intervention (*P *<.001)
